# Department of Practice of Medicine

**Published:** 1893-05

**Authors:** Allen J. Smith

**Affiliations:** Medical Department University of Texas, Galveston


### DEPARTMENT OF PRACTICE OF MEDICINE.

Conducted by Professor Allen J. Smith, A. M., M. D., Medical Department University of Texas, Galveston.

---

THE SHURLEY-GIBBS TREATMENT OF TUBERCULOSIS.—Dr. E. Fletcher Ingalls, of Chicago (*Lanphear's Kansas City Medical Index*, January, 1893), gives an analysis of forty-two cases of tuberculosis, treated by himself according to the iodine and sodium and gold treatment, more commonly known as the Shurley-Gibbs treatment, the following: These cases were all cases of pulmonary tuberculosis, treated in this manner for a period of five weeks or more, without regard to the origin, stage, course or result of the disease. Such cases, the progress of which seems to have been checked for several months, in which the general health continues good, and the cough and other symptoms have nearly or quite disappeared, are classified as arrested. The treatment has not seemed to destroy or change the appearances of the tubercule bacilli in those cases in which the germs were sought. No cases are classed as cured, as the author holds that no patient can be properly counted cured, in a disease like pulmonary tuberculosis, who has not been free from all symptoms and signs for at least two years.

Of these forty-two cases, fourteen (or one-third) received no apparent benefit; but twelve of these fourteen were far advanced when they came under treatment. Fourteen others were apparently decidedly improved for some time, but ultimately grew worse, and six have since died. Of the fourteen first mentioned, all are dead, according to the author's belief. Six of the forty-two are noted much improved; all have been under observation for many months, and are, with one exception, doing well. Four of these had been ill but a few months, and were in the early stages. Eight cases of the forty-two are noted as arrested, all of whom are in good health, although bacilli and expectoration continue in small amount every day.

Dr. Ingalls believes the method of decided promise in the treatment of incipient cases, but would not rely on it alone, adding to it all the beneficial elements of the routine treatments.

---

ALBUMINURIA IN THE APPARENTLY HEALTHY.—W. H. Washburn, of Milwaukee, contributes to the *Medical News* of April 1, 1893, an interesting article upon the above subject, in which he analyzes thirty-eight cases of albuminuria, encountered in 1070 urinalyses made in the course of life insurance examinations. Of these thirty-eight cases, two have died from chronic nephritis, one four years, the other three years, after the examination. One died from pulmonary tuberculosis fourteen months after the examination. Six were lost sight of completely. Fourteen are known to be living, and in apparent good health, after intervals of from one to five years after examination; but the condition of their urine is not known. In three of these last cases, cardiac lesions were associated. Two are known to be still albuminuric, one about six months and the other four years after the examination, and not under medical care; and three more are still albuminuric and under care, one for two years, one for eighteen months, and one six months after the examination. Six are known to have recovered.

From his experience with these cases, the author inclines to agree with Talamon and Lecorche, that albuminuria is, in itself, of but little prognostic value, indicating only the existence of a lesion of the glomerular membrane of the kidneys, and nothing as to the degree or profundity of such disease.

---

YELLOW FEVER.—Dr. Joseph Jones, of New Orleans (*St. Louis Medical and Surgical Journal*, February 1, 1893), in a series of lectures before the class of Tulane University announces that from his extended observation of yellow fever in New Orleans during the past 35 or 40 years he is able to make the following conclusions:

(1). Yellow fever is a continued pestilential fever with two well defined stages, the first one of active chemical changes in the blood and organs, and characterized by elevation of temperature and aberrations of the nervous functions, the other one of depression induced by the sedative action of the febrile poison and by profound changes in the blood and certain organs. Yellow fever is a self-limited disease.

(2). The changes in the blood seem to be continuous, the appearance of the stage of depression being simply the result of the constitutional action of the poison developed during the first stage and thereafter. The blood of yellow fever differs from that of malaria, the corpuscles of the former rapidly assuming a crenated shape with minute transudations upon them. In some cases of yellow fever the blood contains small particles possessing vibratory motion. (Dr. Jones here describes a series of experiments and observations on the blood of yellow fever patients, which are, however, almost all invalidated by the fact that there is not eliminated the grave chance of putrefactive changes in the blood employed, the blood being drawn into bottles and examined or used for experimentation sometimes afterwards.)

(3). The maximum of temperature is rapidly attained on the first or second day of the disease, varying with the severity of the attack from 102° to 112°F. in the axilla—then steadily falling; in some fatal cases it rises again toward the end. The fever during this first stage is essentially a temperature elevation due to the increased chemical changes in the blood. Usually there is an increase of excrementitious solids, but this is not invariable. Neither the rise nor the rapid decline are due to the nervous effects of the poison, probably; since the progress of the case seems to bear a more or less definite relation to the degree of hæmic change.

